# Accessibility of Biologic Drugs in Morocco: Comparison with FDA and EMA Approvals (2015–2025)

**DOI:** 10.3390/jmahp14020021

**Published:** 2026-04-09

**Authors:** Mounir Charrak, Yahia Cherrah, Samira Serragui

**Affiliations:** Pharmaco-Epidemiology and Pharmacoeconomics Research Team, Laboratory of Pharmacology and Toxicology, Faculty of Medicine and Pharmacy, Mohammed V University of Rabat, Rabat 10000, Morocco

**Keywords:** biological drugs, accessibility, availability, reimbursement, delays, Morocco, FDA, EMA

## Abstract

This study aims to evaluate the rates and timeframes of the availability and reimbursement of biologic drugs in Morocco, after approval by the Food and Drug Administration (FDA) or the European Medicines Agency (EMA). The results will help to identify disparities in access and promote rapid access to these innovative treatments. This descriptive study established an international reference list of biological medicines, based on data from the FDA and EMA for the period from 2015 to 2025. An analysis was conducted using national sources, focusing on the availability, reimbursement rates, and timeframes for each listed biological drug. Of the 233 listed biological drugs, only 13.7% (32/233) of those approved between 2015 and 2025 are available in Morocco. Of these, 87.5% (28/32) have been priced, and only 10.7% (3/28) have been approved for reimbursement. The average time between FDA/EMA approval and pricing in Morocco is 3.75 years and 3.41 years, respectively, while the average reimbursement approval time is 2.74 years. This study highlights the delay and limited access for Moroccan patients to internationally approved biologic drugs.

## 1. Introduction

Biological drugs have transformed the treatment of many serious diseases, particularly autoimmune, endocrine, cancer, and neurodegenerative diseases, offering therapeutic options where conventional medicine was previously less effective [1].

New biological drugs are approved each year in the United States and Europe. However, there are significant accessibility issues in Morocco due to the high cost and the complexity inherent in their manufacture [1,2,3,4,5].

The delay between the first marketing authorization (MA) of a drug anywhere in the world, usually in the United States and Europe, and its authorization in another country is commonly referred to as the “drug lag” [6]. Patients in countries with significant drug lag experience delayed access to innovative medicines, which has serious consequences for their health, especially for those suffering from life-threatening diseases.

In Morocco, access to drugs involves several sequential regulatory and institutional steps. MA is granted at the national level by the Moroccan Agency for Medicines and Health Products (AMMPS), following a formal application submitted by the manufacturer or the MA holder. Approval by the Food and Drug Administration (FDA) or the European Medicines Agency (EMA) does not automatically result in authorization in Morocco, although it may serve as supporting evidence in the national evaluation process. After registration, the medicine undergoes a separate pricing procedure under the national pharmaceutical pricing framework, also managed by the AMMPS, through which an official public selling price is determined. Reimbursement represents a further and distinct stage, involving the National Health Insurance Agency (ANAM).

Biologic medicines are less available and less frequently reimbursed in Low- and Middle-Income Countries (LMICs) than in high-income countries [7,8,9,10]. In Central and Eastern Europe, only 1–5% of patients with rheumatoid arthritis receive biologic therapy, whereas in Asia-Pacific LMICs, biologic use is extremely low [10,11]. Access also experiences delays: the interval from approval to availability or reimbursement can last several years, with additional delays due to administrative hurdles, regulatory bottlenecks, and limited health system infrastructure [7,12,13,14].

Studies show limited and delayed access to innovative medicines in Morocco. In 2024, a study showed that cancer drugs were introduced between 2 and 7 years after approval by the FDA, and that 22 of the 39 drugs studied were not reimbursed. The average reimbursement approval delay was four years [15]. According to IQVIA, only 66 of the 433 drugs approved by the FDA and the EMA (2010–2021) have been registered in Morocco, including 37 between 2018 and 2021. The average time to registration and reimbursement approval was 2.5 years and 8.1 months, respectively [16]. In 2025, IQVIA reported that out of 593 global innovations (2013–2022), only 68 were approved, and none were reimbursed, confirming the limited accessibility in Morocco [17]. However, these previous studies examined medicines in general and did not specifically focus on biologic drugs. This gap in the literature limits understanding of the barriers to access to biologic drugs in the Moroccan context and hinders the development of evidence-based pharmaceutical policies. It is therefore essential to assess more precisely the status of biologic drugs in Morocco.

The aim of this study was to determine the availability, reimbursement rates, and access timelines of biologic drugs in Morocco, in comparison with those approved by the FDA and EMA between 2015 and 2025. This study focused on originator biologic medicines and excluded biosimilars, vaccines, gene and cell therapies, blood products, and allergens because of their distinct regulatory and clinical frameworks.

The remainder of the paper presents the materials and methods, followed by the results, discussion, and conclusion.

## 2. Materials and Methods

Our study takes a descriptive approach to assess the accessibility of biologic drugs in Morocco over a ten-year period, compared to those approved by the FDA and EMA.

The scope of the study includes new reference biologic drugs that obtained MA from the FDA’s Center for Drug Evaluation and Research (CDER) and the EMA.

In order to compile an international reference list of biological medicines, the FDA and EMA online databases on approvals of reference biological medicines were consulted, covering the period from 1 January 2015 to 31 October 2025. The databases were consulted in November 2025. This approach was chosen to provide an up-to-date overview of internationally approved biologic drugs and their availability in Morocco at a defined reference date.

All substances falling within the definition of biotherapy were included in these approvals, with the exception of biosimilars, gene and cell therapies, vaccines, blood products, and allergens, these categories were excluded due to their distinct regulatory pathways, clinical indications and manufacturing complexities, which would introduce heterogeneity into the analysis of reference biologics.

Each item on the list includes, at a minimum, the international nonproprietary name (INN) of the biological drug in question, its trade name, if applicable, and the date of FDA or EMA approval, expressed as the day, month, and year. When certain INNs have been approved under various trade names, the one that received the first approval was retained [18,19].

A search by name was conducted on the website of the AMMPS for each biological medicine listed by the FDA and EMA, in order to verify whether MA and a price had been granted in Morocco. The results of this analysis made it possible to quantify the regulatory availability rate of international biological medicines on the Moroccan market [20].

The online platform of the ANAM was used to verify the reimbursement status of biological medicines in Morocco [21].

The official bulletins (O.B.) of the Kingdom of Morocco were the reference source for collecting the official dates for setting prices and registering on the list of reimbursable drugs [22].

Several indicators were derived from the data collected in order to meet the objectives of this study:

- Availability rate: this corresponds to the proportion, expressed as a rate and percentage, of reference biological medicines approved by the FDA or EMA between 2015 and 2025, which have obtained MA in Morocco by the date of verification.
- Time to international availability vs. Morocco: this is the interval between the date of initial approval by the FDA or EMA and the date of price setting in Morocco.
- Reimbursement rate: This is the rate and percentage of biologic drugs included on the list of reimbursable drugs.
- Reimbursement period: this is the time between the date the price is set in Morocco and the date of reimbursement approval in the Moroccan system.

Each time period is determined based on specific dates (day, month, year). The difference is initially quantified in terms of the number of days and then converted into an expression in years to facilitate interpretation.

The data were consolidated in Microsoft^®^ Excel^®^ (Microsoft 365 MSO (Version 2511 Build 16.0.19426.20118)) and analyzed using a descriptive statistical tool. The automation of the time calculation was implemented in Excel by subtracting the relevant dates, with manual verification of special cases, in order to obtain an accurate measurement in days and then converting it into interpretable calendar units (years).

The results were expressed as rates and percentages for proportions (MA approval rate, fixed price rate, reimbursement rate), with the absolute number of biological drugs corresponding to each category indicated. With regard to delays, measures of the central tendency, including the mean and median, were calculated to provide an overall indication of the duration of access and the range of delays (minimum and maximum), illustrating the variability between drugs. The tool used for statistical analysis was Jamovi (2024) (Jamovi. Version 2.6) [Computer Software], retrieved from https://www.jamovi.org (accessed on 1 November 2025).

## 3. Results

### 3.1. Biological Drugs Approved by the FDA and EMA

In total, 498 drugs were approved by the FDA between 1 January 2015 and 31 October 2025, according to CDER data, of which 172 (34.5%) were biologics.

During the same period, the EMA approved a total of 655 medicines, of which 193 (29.5%) were biological.

**Figure 1:**
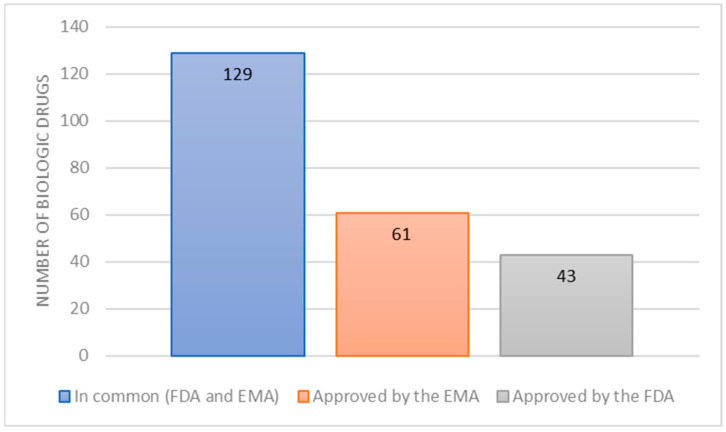
Biological medicines approved by the European Medicines Agency (EMA) and the Food and Drug Administration (FDA) between 2015 and 2025.

After removing duplicates where the same biologic received approvals from both agencies, a total of 233 unique reference biologics were identified (Figure 1).

The full list of the 233 unique biologics included in this study is provided in Table S1: Dataset of the 233 biologic medicines analyzed in the Supplementary Materials.

### 3.2. Availability Rate of Biological Medicines in Morocco

**Figure 2:**
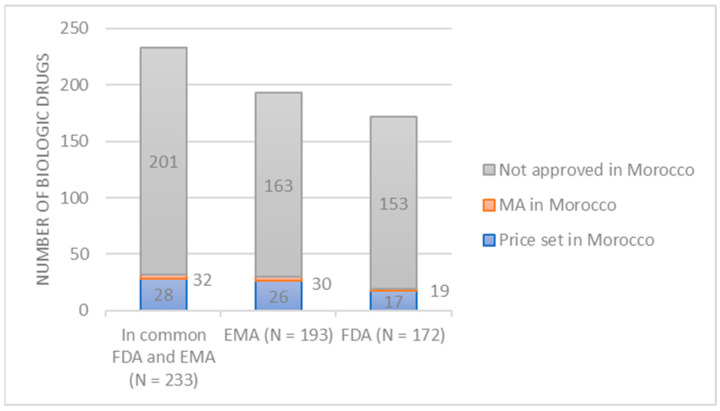
The number of biologic drugs with marketing authorization (MA) and their prices in Morocco compared to the European Medicines Agency (EMA) and the Food and Drug Administration (FDA).

Of the 233 biological medicines approved by the EMA or FDA, 32 (13.7%) had MA in Morocco, of which 28 (87.5%) had their prices set (Figure 2).

Of the 172 biological drugs approved by the FDA, only 19 (11%) are authorized in Morocco. Of these, the price of 17 (89.5%) has already been set in Morocco, while the pricing of the remaining two drugs (10.5%) was still pending at the time of verification (Figure 2).

Only 30 biological medicines (15.5%) out of the 193 approved by the EMA had MA in Morocco, of which 4 (13.3%) were in the process of being priced at the time of verification, while the price of 26 (86.7%) had already been established (Figure 2).

**Figure 3:**
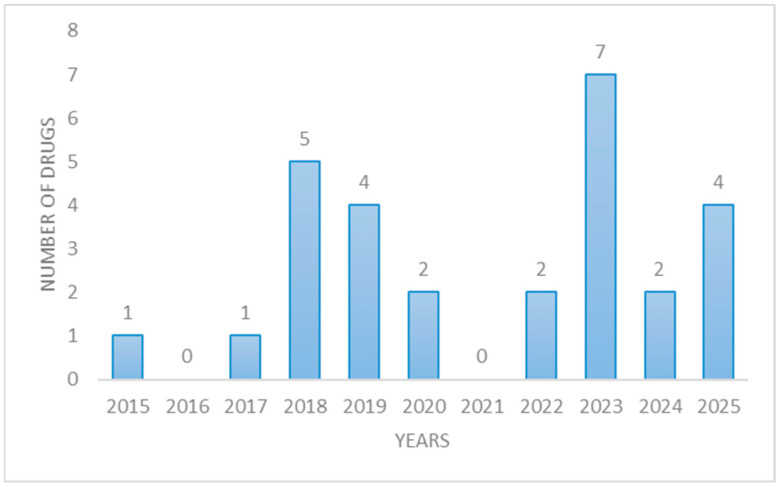
Number of biological drugs whose prices have been set in Morocco.

Between 2015 and 2025, prices were set for 28 biological medicines in Morocco. After an initial phase of moderate growth, there was a substantial increase in 2018 and 2019, followed by a slowdown in the period 2020–2021. Then, there was an increase in 2023 to seven price fixes, which stabilized at a lower level over the period 2024–2025 (Figure 3).

### 3.3. The Timeframe Between FDA/EMA Approval and Pricing in Morocco

The average interval observed between the date of approval by the FDA and the date of pricing of biological drugs in Morocco was 3.75 years. The maximum delay observed was 8.02 years, while the minimum delay was 1.87 years (Table 1).

The time interval between the approval of biological medicines by the EMA and the date on which their price was set in Morocco averaged 3.41 years. This period varied between a maximum of 7.18 years and a minimum of 0.43 years (Table 1).

### 3.4. Reimbursement Rates and Timeframes for Biological Medicines in Morocco

Of the 28 biologic drugs with MA and fixed prices in Morocco, only three (10.7%) were reimbursable.

The average reimbursement period, defined in this study as the interval between the date the price was set in Morocco and the date of reimbursement approval in the Moroccan system, was 2.74 years, ranging from 2.20 to 3.48 years (Table 2).

## 4. Discussion

This study highlighted relatively limited access to biological medicines for Moroccan patients, given that only 13.7% of the internationally approved medicines between 1 January 2015 and 31 October 2025 are available in Morocco, of which 87.5% have been priced, and only 10.71% are reimbursable.

Comparing the date of approval by the EMA or the FDA with the date of entry into the national market is an important indicator of patient access to innovative treatments. The study highlighted the delayed access of Moroccan patients to biological medicines, characterized by an average delay of 3.75 years between the date of international approval by the FDA and the setting of the price in Morocco, with 3.41 years for the EMA.

The average time until reimbursement approval is 2.74 years. Consequently, it appears that a Moroccan patient must wait an average of more than three years to access an internationally approved biological medicine and more than two years to receive reimbursement for it.

Our observations are consistent with previous studies reporting low availability rates and substantial delays in access to new therapeutic agents in Morocco. However, none of these studies specifically examined biologic drugs. Our findings also align with international evidence that indicates that limited and delayed access to biologic medicines is a common challenge across LMICs, not unique to Morocco. Studies report major inequities in reimbursement and use, including very low biologic uptake in Central and Eastern Europe and markedly lower consumption in lower-middle-income Asia-Pacific economies than in high-income countries. Market entry and reimbursement are also often delayed by administrative, financial, and regulatory barriers [7,8,9,10,11,12,13,14].

In this study, the absence of a biologic drug from the Moroccan regulatory or reimbursement system should not be interpreted as evidence of formal rejection. Rather, it indicates that at the reference date, the product was not identified as having obtained MA, price-setting, or reimbursement listing in publicly accessible sources. This may reflect different situations, including non-submission by the manufacturer, ongoing review, absence of price-setting, lack of a reimbursement application, or rejection, which could not be distinguished in the present study. Further research is needed to explore the factors associated with non-availability, non-reimbursement, and prolonged access timelines for biologic drugs in Morocco.

The rates and delays observed in access to biologic medicines in Morocco are attributable to a combination of factors, including economic and regulatory factors.

Pharmaceutical companies favor profitable markets over low- and middle-income countries such as Morocco due to anticipated prices, sales volumes, patents, costs, and low consumption, which limits the national attractiveness [23,24,25].

Companies often delay launching in emerging markets due to patients’ low purchasing capacity and to avoid price erosion in high-value markets [25]. The delay in launching in emerging markets can also be explained by demand: limited financial resources, insufficient infrastructure, cost control, and a lack of expertise in pharmacoeconomic analysis [25,26].

In addition to cost control, access to emerging markets is hampered by regulatory complexity, the priority given to generics, local support, and political instability [26].

The 2015 MA reform increased transparency but did not stimulate approvals. Long delays limit market access, stifle competition, and keep prices high, reducing the expected effectiveness of the reform [27].

Analysis of the price-setting process shows that the regulatory deadline of 60 days is significantly exceeded: delays range from 30 to 260 days in internal committees, from 52 to 274 days in interministerial committees, and up to 339 days for publication in the O.B. [28].

The lack of reimbursement for biologic drugs limits their affordability despite their availability. High costs and the absence of a national reimbursement policy tailored to these drugs lead to inequalities and varying delays in access for patients.

Despite the lengthy market access delays for biological medicines in Morocco, the temporary authorization for use (TAU) mechanism helps to mitigate this delay. This regulatory exemption allows patients early access to innovative biological treatments before they are approved for marketing, particularly for serious or rare conditions for which there is no alternative treatment. As a result, the TAU helps to reduce the negative consequences of regulatory delays on patient management [29]. The long reimbursement delays in Morocco are mitigated by a mechanism allowing early access to reimbursement, sometimes even before MA is obtained, illustrating the national desire to promote therapeutic innovation [30].

Morocco’s healthcare system is undergoing a structural overhaul aimed at strengthening healthcare sovereignty and equitable access to care. This is illustrated by the creation of the AMMPS, responsible for ensuring the availability, quality, and accessibility of health products, as well as the creation of the High Authority for Health, which oversees the evaluation and improvement of the accessibility of medicines, in particular through the development of reimbursement guidelines [31,32,33].

There were five limitations to this study. First, although the study was methodologically rigorous, it remains descriptive. Therefore, it cannot establish causal relationships between variables or precisely identify the mechanisms responsible for the delays observed. Second, the sample of biological medicines examined was limited due to the exclusion of biosimilars, gene and cell therapies, vaccines, blood products, and allergens, because of their specific regulatory frameworks and clinical characteristics, which differ from those of other biological medicines. Their inclusion would have created methodological heterogeneity and biased the comparison between international approvals and access in Morocco. Consequently, the results must be interpreted with caution. Third, the pricing date rather than the MA date in Morocco was used, since there is no official publication of the latter for newly approved drugs. Fourth, a possible limitation of this study is that biologic drugs approved by the FDA or EMA very late in 2025 may not have had sufficient time to obtain MA in Morocco by October 2025, potentially leading to an underestimation of the observed availability rate. Finally, taking into account international approvals from the FDA and EMA facilitates temporal comparability, but it risks overestimating delays if manufacturers did not submit their applications early in Morocco.

Nevertheless, our analysis provides an overall assessment of the rates and timeframes for the availability and reimbursement of biological medicines in Morocco, compared with those of the FDA and EMA over 10 years, based on official data sources, which ensures the accuracy of the information relating to availability and reimbursement status in Morocco. The study proposes specific and quantifiable measures of rates and timeframes, allowing for subsequent comparison with other countries and contexts.

Further investigations should be conducted to identify the factors associated with the rates and delays observed and to compare Morocco with a group of reference countries in order to determine whether the rates and delays observed are specific to Morocco or whether they are a regional feature of health systems. The impact of public health policies on changes in delays and rates of availability and reimbursement should also be examined.

## 5. Conclusions

This study aimed to assess the availability, reimbursement status, and access timelines of biologic drugs in Morocco, in comparison with those approved by the FDA and EMA between 2015 and 2025. The findings indicate that access to biologic drugs in Morocco remains constrained by delays across successive market access stages, consistent with patterns observed in other LMICs. These results provide new evidence on biologic drug accessibility in Morocco and may help inform future pharmaceutical policy and research on barriers to timely access.

## Figures and Tables

**Table 1:** Time between Food and Drug Administration (FDA) and European Medicines Agency (EMA) approval and pricing in Morocco.

	Delay in Years Compared to the FDA	Delay in Years Compared to the EMA
N	17	26
Average	3.75	3.41
Median	2.86	2.77
Standard deviation	1.93	2.00
Minimum	1.87	0.430
Maximum	8.02	7.18

**Table 2:** Reimbursement rates and time-to-reimbursement for biologic drugs in Morocco.

	Delay in Years
N	3
Average	2.74
Median	2.54
Standard deviation	0.660
Minimum	2.20
Maximum	3.48

## Data Availability

The data presented in this study are openly available from publicly accessible sources, namely the U.S. Food and Drug Administration (FDA) and the European Medicines Agency (EMA) official websites. Full citations and links to the original data sources are provided in the reference section of the manuscript. The full list of the 233 unique biologics included in this study is provided in Table S1: Dataset of the 233 biologic medicines analyzed in the Supplementary Materials.

## Supplementary Materials

The following supporting information can be downloaded at: https://www.mdpi.com/article/10.3390/jmahp14020021/s1, Table S1: Dataset of the 233 biologic medicines analyzed.

## Abbreviations

The following abbreviations are used in this manuscript:

MA	Marketing Authorization
FDA	Food and Drug Administration
EMA	European Medicines Agency
CDER	Center for Drug Evaluation and Research
INN	International Nonproprietary Name
AMMPS	Moroccan Agency for Medicines and Health Products
ANAM	National Health Insurance Agency
O.B.	Official Bulletins
TAU	Temporary Authorization for Use
LMIC	Low- and Middle-Income Countries
